# Computer Vision Based Method and System for Online Measurement of Geometric Parameters of Train Wheel Sets

**DOI:** 10.3390/s120100334

**Published:** 2011-12-30

**Authors:** Zhi-Feng Zhang, Zhan Gao, Yuan-Yuan Liu, Feng-Chun Jiang, Yan-Li Yang, Yu-Fen Ren, Hong-Jun Yang, Kun Yang, Xiao-Dong Zhang

**Affiliations:** 1 Department of Physics, Zhengzhou University of Light Industry, Zhengzhou, 450002, China; E-Mails: fengchunj@zzuli.edu.cn (F.-C.J.); yangyanli@163.com (Y.-L.Y.); r_yufen@163.com (Y.-F.R.); theredarmyhust@126.com (H.-J.Y.); yyyk2002@163.com (K.Y.); zhangxiaodong@zzuli.edu.cn (X.-D.Z.); 2 Key Laboratory of Luminescence and Optical Information of Ministry of Education, Beijing Jiaotong University, Beijing, 100044, China; E-Mail: zhangao@bjtu.edu.cn; 3 Library of Zhengzhou University of Light Industry, Zhengzhou, 450002, China; E-Mail: yuanyuanliu83@gmail.com

**Keywords:** wheel set, calibration, computer vision, optoelectronic technique, mapping function method

## Abstract

Train wheel sets must be periodically inspected for possible or actual premature failures and it is very significant to record the wear history for the full life of utilization of wheel sets. This means that an online measuring system could be of great benefit to overall process control. An online non-contact method for measuring a wheel set’s geometric parameters based on the opto-electronic measuring technique is presented in this paper. A charge coupled device (CCD) camera with a selected optical lens and a frame grabber was used to capture the image of the light profile of the wheel set illuminated by a linear laser. The analogue signals of the image were transformed into corresponding digital grey level values. The ‘mapping function method’ is used to transform an image pixel coordinate to a space coordinate. The images of wheel sets were captured when the train passed through the measuring system. The rim inside thickness and flange thickness were measured and analyzed. The spatial resolution of the whole image capturing system is about 0.33 mm. Theoretic and experimental results show that the online measurement system based on computer vision can meet wheel set measurement requirements.

## 1. Introduction

A railway wheel set is subject to normal wear due in large part to friction contact between the wheel set and the rails [1]. In order to ensure the safety of a running train, it’s very important to detect the status of the wheel set automatically and regularly [2]. When wear is severe, dressing or replacing must be applied to ensure the safety. This means that an online measuring system would be of great benefit to overall process control.

A variety of online techniques for measuring a wheel set’s geometric parameters have previously been discussed in the literature. Feng *et al*. [3] introduced a method to measure wheel set parameters based on laser displacement sensors. Wu *et al*. [4,5] presented theoretical methods to measure wheel sets’ diameters and defects online based on the image processing. Zhang *et al*. [6–8] developed opto-electronic measuring methods based on wavelet analysis denoising. Naumann *et al*. [9] applied some opto-electronic sensors to measure wheel sets’ parameters, including the diameter, flange thickness, rim width, flange height and so on. Mian *et al*. [10] measured wheel sets’ parameters with an opto-electronic system based on displacement sensors. Gomez *et al*. [11] employed some general image processes like dilation, erosion, and thinning to measure wheel sets’ parameters online, however, the measuring system must be mounted on a special rail. Pagano *et al*. [12] presented an ultrasonic real-time inspection system and method, which performed defect recognition on a trail wheel.

The objective of the paper is to utilize the opto-electronic measuring technique to develop a non-contact measurement system capable of measuring online the geometric parameters of wheel sets. In this system, the rim inside thickness and flange thickness can be automatically measured without contact. The remainder of this paper is organized as follows: in Section 2, the proposed measuring principle, system, and calibration are presented. Section 3 describes the experimental results and discussion and conclusions are drawn in Section 4.

## 2. The Proposed System

### 2.1. Measuring Principle

**Figure 1.**
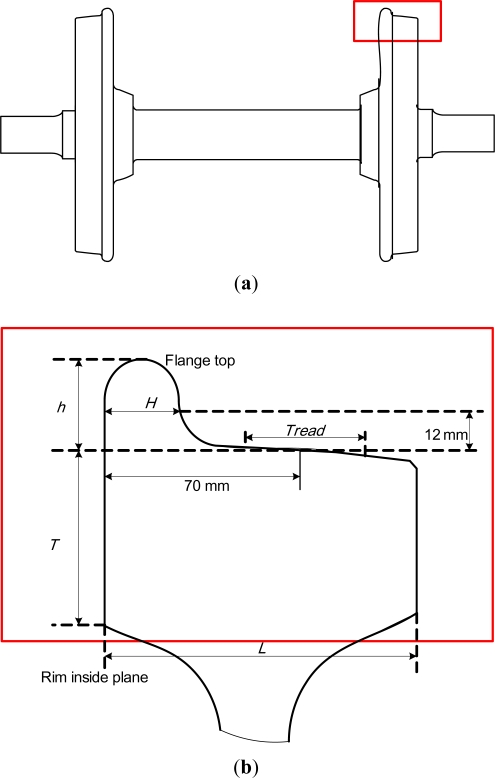
Profile of a wheel set. (**a**) Side view of a wheel set; (**b**) Partial enlargement of a wheel set.

Figure 1 shows the profile of a wheel set. Tread is the contact zone with the rail when the wheel set moves. There is a base point on the tread and the distance to the rim inside plane is 70 mm. Flange base line is the horizontal line 12 mm above the contact point of the wheel set and rail. Flange height, *h*, is the height between flange base line and flange top. Flange thickness, *H*, is the horizontal thickness along the flange base line. After the wheel set has worked for a long time, the actual values of the flange height and thickness will be different from the original ones, which is called flange wear. Rim inside thickness, *T*, is the distance between the flange base line to the bottom of rim inside plane. Rim width is the distance between inside base plane and outside base plane. The rolling circle is a circle passing through the contacting point on the tread and the diameter of the rolling circle is the diameter of the wheel set. These parameters constitute the geometric parameters of the wheel set.

**Figure 2.**
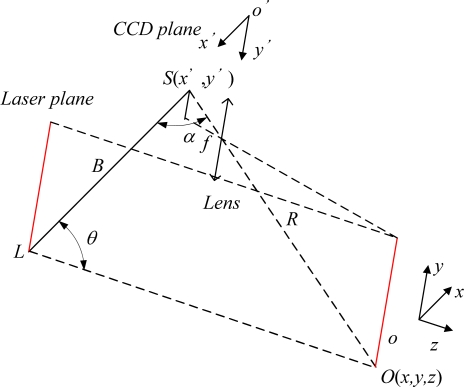
CCD geometry imaging analysis.

The basic principle of profile measurement based on the linear structure lighting is shown in Figure 2. The measurement system is composed of a linear semiconductor laser and CCD camera. The measurement principle is based on the triangulation principle [13]. When the distance (*B*) between two points (*L*, *S*) and the angles (*θ*, *α*) from each of these points to a third point (*O*) are known, the coordinate of the third point can be calculated by the following equation:

(1) $$R=B\frac{\text{sin}\;\theta }{\text{sin}(\alpha +\theta )}$$

where *B* is the baseline distance between two points, and *θ* and *α* are the respective angles.

The angles *θ* and *α* can be geometrically calculated using the coordinates of associated image pixels, respectively. *S* and *L* are the centers of sensor and light source, respectively. The baseline *B* is obtained through the calibration. A point *O* (*x*, *y*, *z*) in 3-D space on the object surface can be calculated through the following equations:

(2) $$x=\frac{B\text{tan}\;\theta }{f-u\cdot \text{tan}\;\theta }\cdot u$$

(3) $$y=\frac{B\text{tan}\;\theta }{f-u\cdot \text{tan}\;\theta }\cdot v$$

(4) $$z=\frac{B\text{tan}\;\theta }{f-u\cdot \text{tan}\;\theta }\cdot f$$

where *u* and *v* are the pixel coordinates of the sensor image, and *f* is the focal length of the CCD camera.

### 2.2. Measuring System’s Composition

**Figure 3.**
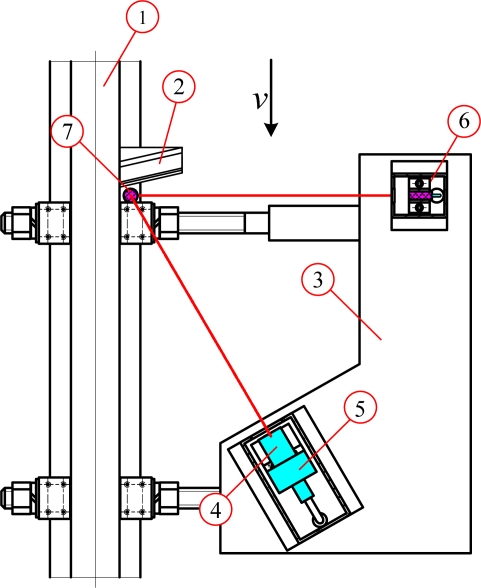
Measurement system schematic diagrams. (1) Rail. (2) Reflector. (3) Fixing table. (4) Lens. (5) CCD camera. (6) Linear laser-1. (7) Linear laser-2.

Figure 3 shows the schematic diagram of the measuring system, which consists of the computer vision setup, two linear lasers (R635UL5-3, Xi’an Ruichen Optoelectronic Technology Co., LTD., China), and the reflector (Daheng New Epoch Technology, Inc., China). In computer vision setup, the grayscale images are captured through CCD camera (OK_AM1100, Beijing JoinHope Image Tech. Ltd., China) equipped with the 16-mm industrial lens (M1614-MP, CBC Beijing Co. Ltd., China). The signal is digitized into the computer memory through a PCI frame capture board (OK_M10A, Beijing JoinHope Image Tech. Ltd., China).

**Figure 4.**
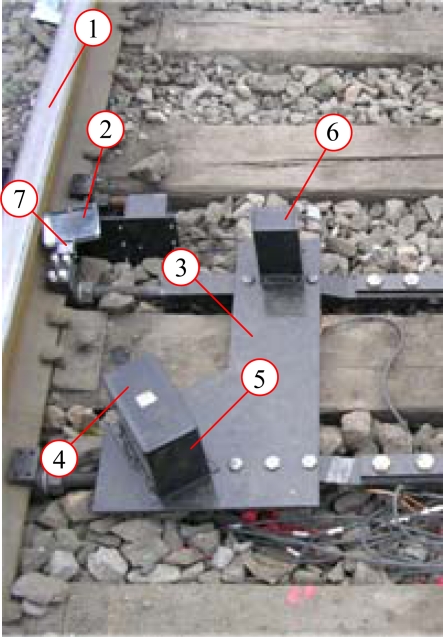
Wheel set image measurement system in the field.

The wheel set image measurement system in the field is shown in Figure 4. The CCD camera and the linear laser were protected by the safety cover with a window. The measuring system was fixed to the rail by the fixing table to avoid the effects from vibration and hunting motion of wheel sets.

Clear image acquisition is very important to ensure high measuring accuracy. The relative positions of the light source, CCD camera and wheel detecting sensor are very important. The image quality increases as the resolution increases, however, the cost and the required memory capacity also increases. Since only the grey level variation is considered for image analysis, a black and white camera was selected and the specifications are shown in Table 1. The exposure time of the CCD camera is set to 1/10,000 second and the image acquisition mode uses the asynchronous reset function.

**Figure 5.**
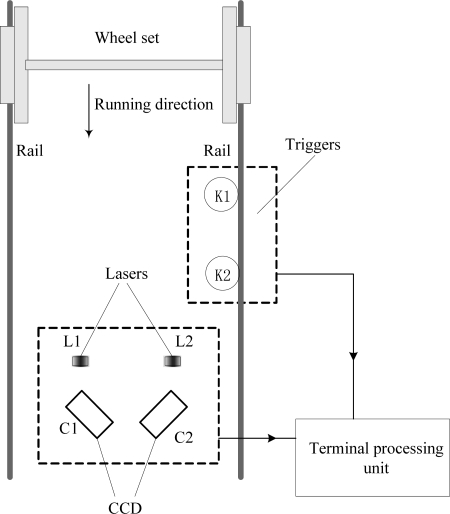
Schematic diagram of measurement system.

**Figure 6.**
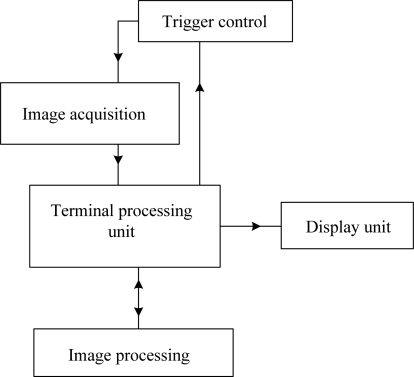
Image processing flowchart.

Figure 5 shows a schematic diagram of the measurement system, which includes wheel detecting sensors (K1, and K2), and linear lasers (L1, and L2), and CCD cameras (C1, and C2), and lens, and optical filters, and terminal processing unit. When wheel sets pass the triggers (wheel detecting sensors) the triggers start the measurement system. The speed (*v*) of a train passing through the measurement system can be obtained by wheel detecting sensors. The distance (*l*) is measured between the wheel detecting sensor and the CCD camera. The camera delay time was obtained using the equation *t* = *l*/*v*. The images captured by the CCD cameras are transmitted to the terminal processing unit. Figure 6 shows the flowchart of the image processing. The initial data and measuring results are transferred to a database to compare and maintain wheel sets.

### 2.3. System Calibration and Image Processing

**Figure 7.**
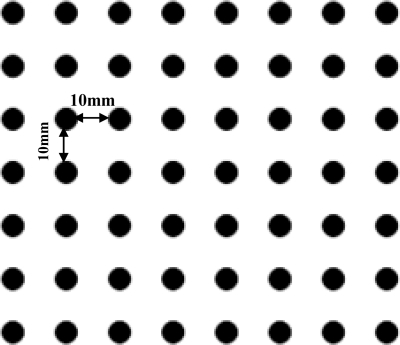
Coplanar reference target.

**Figure 8.**
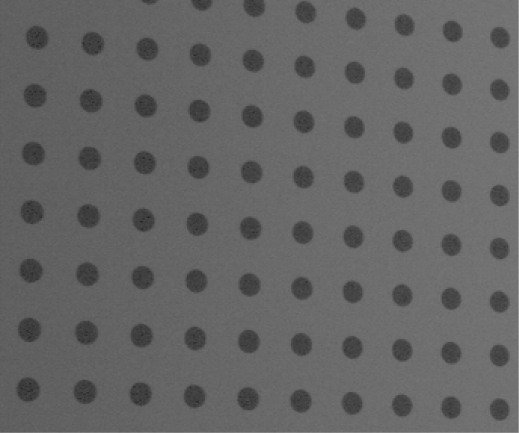
Target’s image captured by the CCD.

The CCD captured image would be distorted and lead to measurement errors because of lens distortion. Therefore, the CCD camera parameters must be calibrated before use. A CCD camera includes two sets of parameters: extrinsic parameters, and intrinsic parameters. In this research, a speedy method to calibrate the CCD camera is established. The mapping function method presented by Dai [14] was adapted to derive the relationship between the measuring plane spatial coordinate and CCD camera image coordinate. The coplanar reference target, as shown in Figure 7, consists of black circles with a diameter of 1 mm distributed regularly at 10 mm intervals. The calibration process was divided into the following main stages:

In the first stage, the laser light plane and target was adjusted in the same plane before calibration and the target was full-field in the camera. The target’s image captured by the CCD is shown in Figure 8.

**Figure 9.**
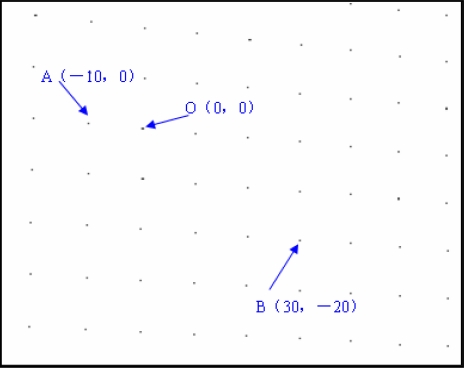
Center coordinates of black circles extraction.

The second stage of work focused on extracting the center coordinates of the black circles. Figure 9 shows this stage. A random point was viewed as the origin *O* (0, 0) in the spatial coordinate and then other points’ coordinates can be confirmed, such as *A* (−10, 0), and *B* (30, −20). Second-order mapping functions that will be obtained with regression analysis. The mapping function is shown as follows:

$$\begin{array}{l} X=a_{1}u^{2}+b_{1}\mathrm{uv}+c_{1}v^{2}+d_{1}u+e_{1}v+f_{1}\\ Y=a_{2}u^{2}+b_{2}\mathrm{uv}+c_{2}v^{2}+d_{2}u+e_{2}v+f_{2} \end{array}$$

where (*X*, *Y*) is the spatial coordinate (unit: mm); (*u*, *v*) is the corresponding image coordinate (unit: pixel).

The derived coefficients after calibration are as follows:

$$\begin{array}{ll} a_{1}=0.00000 & a_{2}=0.00000\\ b_{1}=0.00000 & b_{2}=0.00000\\ c_{1}=0.00000 & c_{2}=0.00000\\ d_{1}=2.45000 & d_{2}=0.11000\\ e_{1}=0.06000 & e_{2}=2.54000\\ f_{1}=-213.25 & f_{2}=-123.32 \end{array}$$

**Figure 10.**
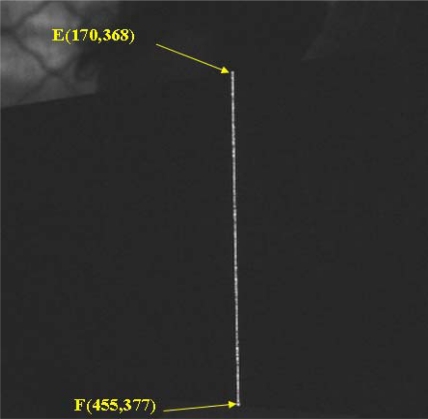
Image of the standard block.

In the third stage, the laser strip was projected on the standard metal block (80 × 50 × 20 mm) and the image shown in Figure 10 was captured by the CCD. The length of the laser strip, the white line (*EF*), was the image coordinate of the length of the block.

In the fourth stage, the start point *E* (170, 368) and end point *F* (455, 377) of the laser strip in the image coordinate were converted to the spatial coordinates, *E*’ (14.48, −4.02) and *F*’ (14.48, −84.25). The length of standard metal block can be obtained, *L*= 80.23 mm. Then three repeated measuring results with different positions are shown in Table 2. The comparison results with the vernier caliper show an error range is ±0.20 mm.

**Figure 11.**
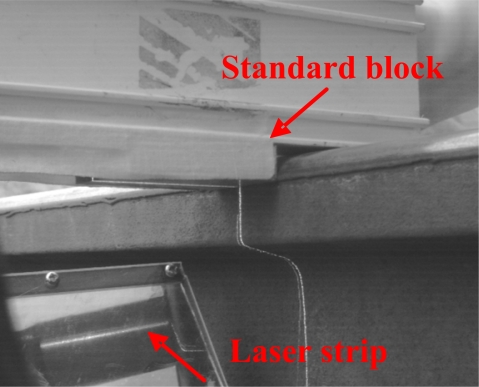
Calibration image of flange thickness in the field.

The final stage of work focused on measurement of obtained pixels in spatial unit transformations about the measuring flange thickness. According to the definition of flange thickness, we designed a calibration bar. The top surface of the rail is considered as the calibration baseline. A standard metal block (55 × 20 × 12 mm) is fixed on the bar with the smooth surface, which is placed on the top surface of the rail. The laser strip is projected on the standard metal block and the image in the reflector shown in Figure 11 is captured by CCD.

The practical distance was 500 mm between the lens and measured object, so the lens field of view is 25 cm × 1.9 cm, and the spatial resolution of the image card is 768 × 576. The spatial resolution of the whole image capturing system is as follows:

dX=250/768=0.33 mm

dY=19/576=0.33 mm

## 3. Results and Discussion

Table 3 shows the multiple measurement results of rim inside thickness and flange thickness of normal wheel sets. The rim inside thickness measuring mean of the vision system is 51.427 mm, and the manual one is 51.577 mm. The standard deviation is 0.032 mm and 0.082 mm, respectively. The difference between the system measurement and manual measurement is within ±0.40 mm. To the flange thickness measurement of wheel sets, the measuring mean of the vision system is 28.375 mm, and the manual one is 28.448 mm. The standard deviation is 0.121 mm and 0.293 mm, respectively. The difference between the system measurement and manual measurement is within ±1.0 mm. The measurement results’ deviation comes from the following reasons:

Firstly, there are different base points for different operators, which will lead to the measuring errors.

Secondly, there are gross errors, such as 51.81 mm and 29.38 mm, in the manual measurement results because of operator mistakes. For the rim inside thickness measurement, when the gross error is removed, the mean and standard deviation is 51.551 mm and 0.009 mm, respectively. For the flange thickness, the mean and standard deviation is 28.344 mm and 0.028 mm, respectively after the gross error removal.

Thirdly, the measurement mode is different. The manual measurement is offline and the system is online, so the manual measurement precision is prior to the system one. In the measurement system based on computer vision, the measurement accuracy is mainly influenced by the following factors: optical system error, wheel vibration error, triggers error, movement error and so on.

In the first place, the measurement systems are mounted on the rail, so the wheel vibration error can be neglected;

Secondly, the optical system error is mostly determined by the CCD camera resolution, Δ*H*_CCD_ = 0.23 mm, and the image processing error is within 2 pixels, Δ*H*_processing_ = 0.46 mm;

Thirdly, when the running speed of wheel sets is 5 km/h, and the exposure time is set to 1/10,000 s, the movement error is 0.14 mm, Δ*H*_movement_ = 0.14 mm;

Finally, the error of trigger is within 0.10 mm. Then, the maximum theoretical error can be obtained [4]:

$${\Delta H}_{\mathrm{Total}}=\sqrt{{\Delta H}_{\mathrm{CCD}}^{2}+{\Delta H}_{\mathrm{Processing}}^{2}+{\Delta H}_{\mathrm{Movement}}^{2}+0.1}=0.54\;\mathrm{mm}$$

## 4. Conclusions

An on-line non-contact method for measuring the wheel sets’ parameters is presented based on the machine vision technique. CCD cameras with a selected optical lens and a frame grabber are used to capture the image of the light profile of the wheel set illuminated by a linear laser. The analogue signals of the image were transformed into corresponding digital grey level values. The ‘mapping function method’ is used to transform an image pixel coordinate to a space coordinate. The images of wheel sets were captured. The rim inside thickness and flange thickness were measured and analysed. Theoretical and experimental results show that the measurement system based on computer vision can meet the wheel set measurement requirements online.

## Figures and Tables

**Table 1.** Main parameters of CCD camera (OK_AM1100).

**Frame Frequency Range (Hz)**	20–70

**CCD Sensor (Inch)**	1/2 inch
**CCD Size (mm)**	7.48 × 6.15
**Pixel Size (um)**	8.3 × 8.3
**Effective Pixels**	768 × 576
**Sensitivity (lux)**	0.05
**SNR (dB)**	>56

**Table 2.** Measurement results’ comparison.

**Measuring order**	1	2	3

**Vernier caliper measurement (mm)**	80.06	80.08	80.08
**Computer vision measurement (mm)**	80.23	80.22	80.24
**Deviation (mm)**	0.17	0.14	0.16

**Table 3.** measuring results of the system and manual.

**Wheel set Number**	**Rim inside thickness (mm)**		**Flange thickness (mm)**	
	**System Measurement**	**Manual Measurement**	**System Measurement**	**Manual Measurement**

**1**	51.41	51.55	28.48	28.32
**2**	51.48	51.54	28.27	28.38
**3**	51.41	51.55	28.23	28.32
**4**	51.44	51.55	28.45	29.38
**5**	51.41	51.54	28.42	28.34
**6**	51.43	51.81	28.27	28.36
**7**	51.37	51.55	28.60	28.30
**8**	51.47	51.56	28.24	28.38
**9**	51.43	51.55	28.41	28.36
**10**	51.42	51.57	28.38	28.34

**Mean value**	51.427	51.577	28.375	28.448

**Standard Deviation**	0.032	0.082	0.121	0.329
